# Socioeconomic and Environmental Predictors of Asthma-Related Mortality

**DOI:** 10.1155/2018/9389570

**Published:** 2018-04-24

**Authors:** Ankita Sinharoy, Shubhajit Mitra, Pritish Mondal

**Affiliations:** ^1^Department of Public Health Sciences, Penn State College of Medicine, Hershey, PA, USA; ^2^University of Texas Medical Branch, Galveston, TX, USA; ^3^Department of Pediatrics, Division of Pediatric Pulmonology, Penn State College of Medicine, Hershey, PA, USA

## Abstract

The prevalence of asthma-related mortality (ARM) varies significantly among different countries, possibly influenced by various socioeconomic and environmental conditions (SEC). In-depth epidemiological research is necessary to understand the causal relationship between different SECs and ARM and to develop public health strategies to reduce the global burden of asthma. Our research aimed to identify the key SECs which may be attributed to ARM worldwide and to study the relationship between ARM and asthma prevalence. We included twenty-two countries with available data on SECs (2014-2015) and divided them into four groups: Asia, Africa, Europe, and Miscellaneous (Australia and North and South America). Tertiary school enrollment (TSE), gross domestic product (GDP), air pollution index, and male and female smoking prevalence rates were analyzed as predictors of ARM, using multiple linear regression. We found that ARM and asthma prevalence had an inverse relationship and developing countries compared to developed countries experienced higher ARM despite having lower asthma prevalence. Asian and African countries, compared to Europe and Miscellaneous countries, experienced poorer SECs, possibly associated with higher ARM. Among SECs, TSE and GDP had strongest association with ARM. In conclusion, lack of education and uneven distribution of resources may have an influence on the increased ARM in developing countries.

## 1. Introduction

Asthma is one of the most prevalent chronic inflammatory airway diseases; it manifests as recurrent wheezing, coughing, and shortness of breath, secondary to airway hyperresponsiveness [1]. World Health Organization (WHO) conducted the last World Health Survey on asthma in the year 2002-2003 and observed its highest prevalence among adults (18–45 years) of Australia, Brazil, and Northern and Western Europe [2]. Primary attributes of asthma-related mortality rate (ARM) in developing and underdeveloped countries are suboptimal long-term medical care, lack of access to the essential drugs, use of nonprescription medication, and delay in obtaining medical care during fatal asthma attack [3–5]. A recent global asthma report indicated a sharp rise in asthma prevalence from 235 million to 334 million between the years of 2011 and 2014. In 2015 alone, 383,000 people died of asthma. An additional burden of 100 million new cases is estimated by the year 2025 [6]. The global asthma report also illustrated a significant discrepancy in ARM between the developed and underdeveloped world [6]. The prevalence of asthma is strikingly different among various parts of the world and may even vary by 20–60-fold in one country compared to the other [7]. Per the global surveillance report on chronic respiratory disease (2007) by WHO, ARM might not have a direct correlation with asthma prevalence [8]. The report also indicated that asthma prevalence was parallel to environmental allergy and urbanization, while the high ARM was likely to be due to poor socioeconomic conditions including lack of access to lifesaving medications. Exposure to an environmental allergen can precipitate life-threatening asthma, and* Alternaria* mold is a known offender [9]. Pollen exposure can induce status asthmaticus, a unique example of which is 2016 thunderstorm-induced asthma in Melbourne resulting in 8500 ER visits and nine deaths precipitated by the allergic cloud of pollen [10].

Accurate estimation of the global burden of asthma is difficult due to the problem of data acquisition from low and middle-income countries [11], and no recent studies have been published citing a causal relationship among the various socioeconomic and environmental conditions (SEC) and ARM. GBD chronic respiratory disease report, 2015 [12], emphasized that, to develop better asthma prevention strategies, ongoing surveys comparing disease burden among different countries are necessary.

The outcome of asthma is influenced by the level of education, as greater awareness helps to seek timely medical attention and better parental health education has a favorable impact on the prognosis of childhood asthma [13, 14]. Eagan et al. have demonstrated a reduction in asthma incidence in subjects with a higher level of education [13]. In contrast, poverty is a hindrance to accessing adequate medical care, thus often leading to poor outcome of the disease, whereas most industrialized countries with a higher gross domestic product (GDP) can afford a better healthcare system. Grant et al. have demonstrated that poor socioeconomic status including low income and a low level of education had a correlation with higher ARM [15].

Control of air pollution is necessary for maintaining better respiratory health. Residents of a city with higher air pollution tend to have increased pulmonary symptoms including cough, dyspnea, and other serious conditions like lung cancer. While developed countries like the USA, Japan, and Germany have lesser air pollution, Asian countries like India and China have had an alarming rise in air pollution in recent years [16]. The rising asthma prevalence in the Asian subcontinent can be relevant in this context, since there may be an association between asthma morbidity and degree of air pollution, as described by Trasande and Thurston [17]. Particulate matters are classified by their size and concentrations (*µ*g/M^3^). PM_2.5_ is a measure of smaller particulate matter, while PM_10_ quantifies coarse particulate matter. Two similar studies by Portnov et al. and Tecer et al. had demonstrated that, among all the particulate matters, PM_10_ was the most important determinant of asthma-related exacerbation and hospital admission [18, 19]. Both active and passive smoking are independent risk factors for the rapid decline in lung function in asthmatics, leading to a higher ARM among asthmatic smokers [20, 21]. Over the last decade, the prevalence of smoking has been decreased globally as a result of an effective antismoking campaign [22]. However, tobacco use is still considerably high among the poor and less educated sections of society. While the prevalence of male smoking is equivalent across the countries, the female smoking rate is diverse and greatly influenced by the cultural background of that society [23].

Based on previously stated information, we hypothesized that (i) the difference in SECs across the world may be attributed to the disparity in ARM among different countries. We further hypothesized that (ii) ARM does not depend on asthma prevalence. Thus, our study aimed at (i) determining the impact of SECs (TSE, GDP, PM_10_, and male and female smoking prevalence rate) on ARM and asthma prevalence and (ii) identifying the correlation between ARM and asthma prevalence comparing the available data from certain selected countries.

## 2. Materials and Methods

### 2.1. Sample Selection

We selected 40 countries using a random selection tool [24]. Of those 40, 22 countries had all the required data points available from 2014-2015 and thus were included in our cross-sectional study (Table 1). The following 18 countries were excluded due to lack of available data: Bhutan, Kosovo, Latvia, Uganda, Kazakhstan, Mozambique, Suriname, Bosnia and Herzegovina, Mauritania, Laos, Cape Verde, Fiji, Somalia, Palau, Togo, Vatican City, Rwanda, and North Korea. We categorized the selected countries into four groups. Eight countries were from Asia, four from Africa, and five from Europe, and five other countries were included as the Miscellaneous group (two countries each from North and South America and Australia) (Table 2).

### 2.2. Data Collection

The following resources were utilized to collect data for the study parameters.

(*1) ARM*. ARM data of the individual countries were obtained from WHO 2014 report [25]. When the mortality data from 2014 was not available, we used the ARM for the closest available year for those countries. ARM was expressed as the number of deaths per 1,00,000 population.

(*2) Asthma Prevalence Rate*. Prevalence data were collected from the Global Initiative for Asthma (GINA) committee report, 2014 [26]. It was expressed as a percentage of the population (%).

(*3) Tertiary School Enrollment (TSE)*. We considered the percentage of the general population with TSE as an indicator of educational achievement, and the data was collected from the World Bank report (2015) [27]. If TSE data for an individual country from 2015 was not available, then the information from the closest year available was used. TSE was expressed as a proportion of the population (%) of those who had accomplished it.

(*4) GDP (per Capita)*. GDP is a reflection of the economic strength of a country and expressed in USD for our study. We used the report published by CIA (Central Intelligence Agency) in 2015 [28].

(*5) PM*_10_. Air pollution data (PM_10_) was obtained from the report published by WHO in 2014. PM_10_ was expressed as *μ*g/M^3^ [18, 29, 30].

(*6) The Smoking Prevalence Rate in Males and Females Aged >15 Years*. The data was extracted from the report published by WHO (2015) [31] and expressed as a proportion of the population (%) who smokes.

### 2.3. Power Estimation and Sample Size Determination

We used G^*∗*^Power for the power estimation of the study [32]. The effects of GDP and TSE on ARM were used as a model for the power estimation of our study. We conducted multiple linear regression, considering ARM as a dependent variable using GDP and TSE as the predictors. The power of the study was 0.96. A minimum sample size of 16 was required to find a statistically significant association between the dependent variable and the predictors [input criteria were set as two tails, 0.95 of power (1 − *β*) and 0.05 of *α*].

### 2.4. Statistical Analysis

We used independent-sample *T*-test to compare the variables between the continents. We conducted multiple linear regression analysis using ARM as the dependent variable and TSE, GDP, PM_10_, and male and female smoking rate as the predictors. We used SPSS (version 24) for statistical analysis [33].

## 3. Results

We presented the data as mean ± SD and considered *p* < 0.05 as significant (*∗*) and *p* < 0.001 as highly significant (*∗∗*). ARM, asthma prevalence, and SECs were not significantly different between selected Asian and African countries and between Europe and the Miscellaneous group. The significant results obtained are as follows.

*(a) ARM*. The highest mortality rate was observed in Africa, followed by the Asian countries (Table 2). ARM in the selected Asian countries was significantly higher than the countries in Europe and in the Miscellaneous group (*p* value of 0.02^*∗*^ and 0.03^*∗*^, resp.). Likewise, ARM in the African countries was higher in comparison to Europe and the Miscellaneous group (*p* value of 0.001^*∗*^ and 0.002^*∗*^, resp.).

*(b) Asthma Prevalence*. The highest prevalence rate was observed in the Miscellaneous group followed by European countries (Table 2). Asian countries had significantly lower asthma prevalence in comparison to Europe and the Miscellaneous group (*p* value of 0.027^*∗*^ and 0.009^*∗*^, resp.).

*(c) TSE*. More than half of the population in Europe and the countries from the Miscellaneous group had the privilege to achieve TSE (Table 2). TSE was significantly lower in the Asian countries in comparison to Europe and the Miscellaneous group (*p* value of 0.013^*∗*^ and 0.025^*∗*^, resp.). Similarly, African countries had lower TSE in comparison to Europe and the Miscellaneous group (*p* value of <0.001^*∗∗*^ and 0.007^*∗*^, resp.).

*(d) GDP per Capita*. The GDP was highest among the selected European countries, which was significantly higher in comparison to the GDP of the Asian and African nations (*p* value of 0.002^*∗*^ and 0.003^*∗*^, resp.).

*(e) Air Pollution*. PM_10_ was the worst in the selected Asian countries (Table 2), which was significantly higher in comparison to Europe and the Miscellaneous group (*p* value of 0.002^*∗*^ and 0.005^*∗*^, resp.).

*(f) Smoking Prevalence Rate*. Asian countries had the highest prevalence of male smoking, which was significantly higher compared to Europe and the Miscellaneous group (*p* value of 0.02^*∗*^ and 0.003^*∗*^, resp.). In contrast, female smoking prevalence was significantly higher in the selected European countries in comparison to the Asian, African, and Miscellaneous countries (*p* value of <0.001^*∗∗*^, 0.001^*∗*^, and 0.013^*∗*^, resp.).

**Figure 1:**
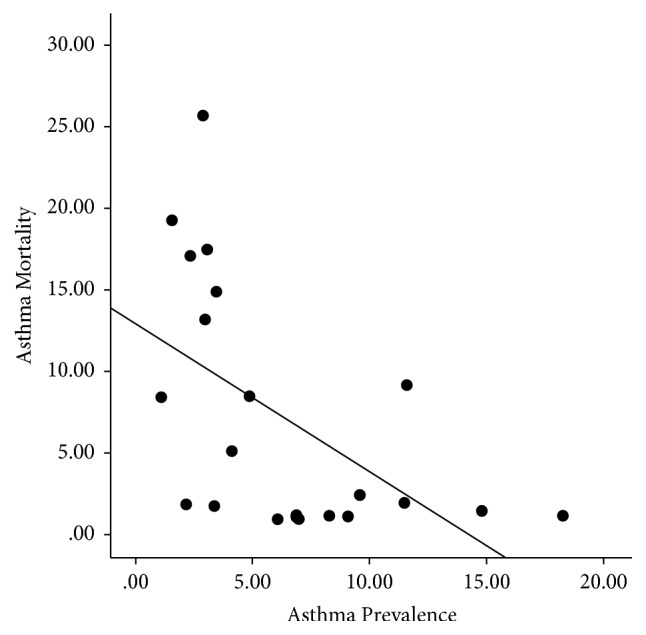
Scattered plot showing a significant negative correlation between asthma mortality rate and asthma prevalence rate.

**Figure 2:**
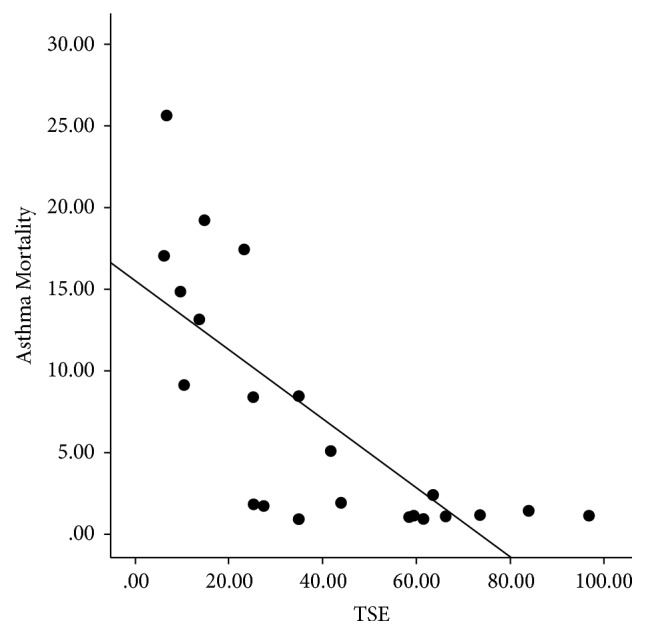
Scattered plot demonstrating a significant positive correlation between TSE and asthma prevalence rate.

**Figure 3:**
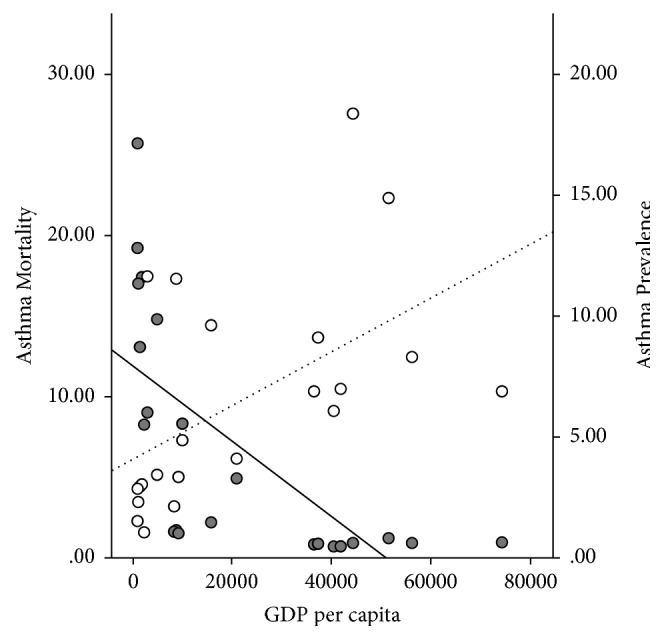
Dual axes scattered plot illustrating the reverse relationship between asthma mortality rate and GDP (marked with the solid dots and solid trend line); and between asthma prevalence rate and GDP (indicated by the transparent dots and dotted trend line).

There was a significant negative correlation between ARM and asthma prevalence [correlation coefficient (*r*) of −0.54, *p* = 0.009^*∗*^] (Figure 1). ARM had strong negative correlation with TSE and GDP (*r* = −0.75 and *p* < 0.001^*∗∗*^ and *r* = −0.68 and *p* = 0.001^*∗*^, resp.) (Table 3). In contrast, asthma prevalence had significant positive correlation with both TSE and GDP (*r* = 0.61, *p* = 0.003^*∗*^ and *r* = 0.54, *p* = 0.01^*∗*^, resp.) (Figures 2 and 3).

We conducted a stepwise multiple linear regression to estimate the degree of influence of predictor variables (TSE, GDP, PM_10_, and male and female smoking prevalence) on ARM. The results indicated that the overall model was statistically significant (*F* = 25.49, *p* < 0.001^*∗*^, CI = −0.30 to −0.12^*∗*^). Furthermore, TSE was the only predictor variable which was statistically significant while adjusted for PM_10_, GDP, and male and female smoking prevalence. Though simple linear regression demonstrated a significant inverse correlation between ARM and GDP based on the *t*-value (−4.1) and *p* value (0.001^*∗*^), multiple linear models excluded GDP due to multicollinearity [high variation inflation factor (VIF) = 4.1].

**Figure 4:**
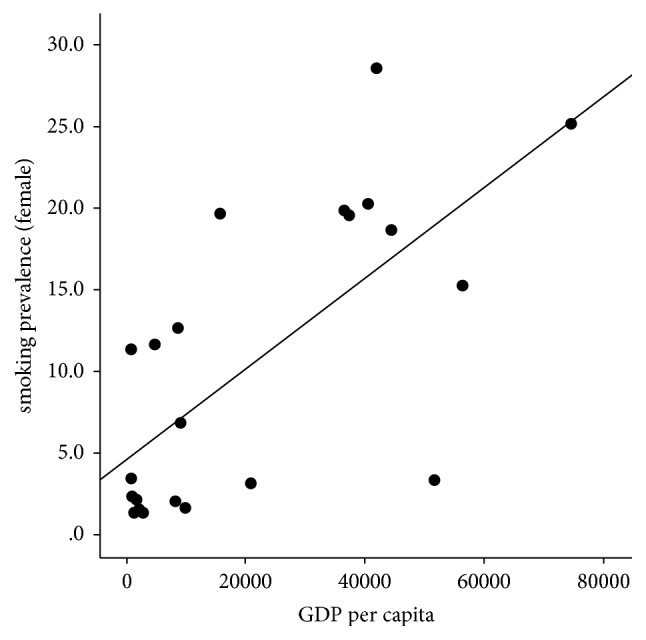
Scattered plot demonstrating the positive correlation between female smoking rate and GDP.

Interestingly, female smoking prevalence, but not male smoking prevalence, had a positive correlation with TSE and GDP (*r* = 0.60, *p* = 0.003^*∗*^ and *r* = 0.69, *p* < 0.001^*∗∗*^) (Figure 4).

## 4. Discussion

Our study demonstrated that selected Asian and African countries have significant poverty, lack of education, and a higher level of air pollution in comparison to European and Miscellaneous countries, which reflected poor living conditions in the developing and underdeveloped countries. ARM had a negative relationship with GDP and TSE. The regression analysis demonstrated that among all the SECs, TSE was likely to be the strongest predictor of the increased ARM, while adjusting for other variables. TSE had a significant association with GDP (*r* = 0.87, *p* < 0.001^*∗∗*^). A strong collinearity demonstrated interconnection between TSE and GDP (VIF of 4.1) and excluded GDP from the multiple regression models. That observation was also supported by the strong association between ARM and GDP in the simple regression model and it was reasonable to also choose GDP as the key predictors of ARM. Thus, our results supported the hypothesis that a few of the SECs possibly contributed to the disparity in ARM. Henceforth, by our study analysis, we inferred that higher asthma-related deaths in Asia and Africa, at least in part, were contributed by poverty and a lack of education.

The inverse correlation between ARM and asthma prevalence helped to substantiate our second hypothesis that increased ARM was not a result of higher asthma prevalence.  Figure 3 highlighted the opposite relationship between GDP and ARM versus GDP and asthma prevalence. This observation was in concordance with previous studies, which cited that, with increased urbanization, the developed countries were experiencing increased prevalence of asthma while having a low ARM [34]. On the contrary, underdeveloped countries, despite having modest prevalence, had significantly high mortality rates [28]. In our opinion, the current evidence is insufficient to explain the global discrepancies between asthma prevalence and ARM and should be the focus of future epidemiological research.

Faniran et al. reported a significantly higher prevalence of asthma in children from Australia, as compared to the children from Nigeria, though the prevalence of atopy was similar between those two countries [35]. The authors also recognized that a substantial proportion of Nigerian children with a wheeze had never been diagnosed with asthma, and many of them never showed up for a follow-up clinic visit. This trend had shown that lack of medical facilities and an inadequate data collection system in the underdeveloped countries could have led to an underestimation of the true prevalence of asthma, which could have explained the positive correlation between lower GDP and decreased asthma prevalence (Figure 3) [36]. In comparison, ARM was far more likely to draw attention and get reported and thus could be considered as a better indicator of the disease burden.

The smoking prevalence among both sexes was equivalent in Europe, while the female smoking compared to the male smoking rate was significantly less in Asia. Countries with higher GDP and TSE had higher female smoking prevalence. It was beyond the scope of this study to investigate the rationale behind that observation. However, different countries had diverse sociocultural practices and religious beliefs, which were often influenced by the level of education and financial freedom in women. The female smoking rate could have been a reflection of that heterogeneity. Future studies should focus on female smoking patterns since maternal smoking significantly influences the outcome of childhood asthma [37].

One of the limitations of this study was the potential chance of introducing selection bias due to lack of data availability. Though the samples were chosen randomly, we could only include those countries with all the available data points. Thus, the likelihood of inclusion of the bigger countries was higher, and our selection might not be a truly global representation. Despite that, epidemiological analysis of this study outlined unquestionable socioeconomic disparities in different parts of the world. In the future, our study hypotheses should be tested with diverse and larger samples as a prospective cohort study. To reduce the global disease burden, the future preventive strategies for asthma should focus on better education and access to the healthcare, and that may help to reduce the gap in asthma-related death between rich and poor countries.

In the age of this ever-increasing burden of asthma, developed and underdeveloped countries need to work collectively to develop a universal strategy to address the problem at multilevel involving healthcare delivery, policy making, and educating people about the disease, under a global platform of cooperation.

## 5. Conclusion

This research enabled us to conclude that, among various socioeconomic and environmental factors studied, only higher GDP and better levels of education are associated with a reduction in asthma-related deaths. Our study also supported the view of global surveillance report (WHO) [8] that asthma prevalence may not directly reflect ARM of a country.

## Figures and Tables

**Table 1:** Country-wise distribution of asthma severity indices and socioeconomic predictors.

Country	ARM (death/100000)	Asthma prevalence (% of population)	TSE (% of population)	GDP ($/capita)	PM_10_ (*µ*g/M^3^)	Male smoking rate	Female smoking rate
Australia	1.20	14.7	83.47	51352	12.7	16.7	3.1
Bangladesh	12.92	2.91	13.3	1208	153.5	43.7	1.1
Belgium	0.69	6.0	34.5	40278	25.8	26.5	20
Brazil	1.69	11.4	43.5	8528	36	21	12.4
China	1.60	2.1	24.87	8109	88	47.6	1.8
France	0.82	6.8	58	36304	24.2	25	19.6
Germany	0.70	6.9	61.06	41686	21.7	32.4	28.3
India	17.20	3.0	22.86	1614	102.1	20.4	1.9
Israel	0.86	9.0	65.8	37129	62.5	41.2	19.3
Malaysia	8.22	4.8	34.5	9768	27	43	1.4
Mali	25.40	2.82	6.34	744	35.9	36.8	3.2
Mexico	1.50	3.3	27.04	8981	61.8	20.8	6.6
Namibia	14.62	3.39	9.3	4674	45.2	38.9	11.4
Nepal	18.99	1.5	14.4	725	114	37.1	11.1
Nigeria	8.90	11.5	10.07	2714	201.9	17.4	1.1
Norway	0.94	6.8	73.1	74186	18.3	25.5	24.9
Saudi	4.86	4.05	41.32	20711	87	27.9	2.9
UK	0.90	18.15	58.99	44162	19.6	19.9	18.4
Uruguay	2.17	9.5	63.1	15574	27	26.7	19.4
USA	0.90	8.2	96.32	56054	16	19.5	15
Vietnam	8.16	1.04	24.8	2068	62	47.1	1.3
Zimbabwe	16.81	2.28	5.8	890	36.9	31.2	2.1

**Table 2:** Continent-wise distribution of socioeconomic predictors of asthma (mean ± SD).

Continent	ARM (death/100000)	Asthma prevalence (% of population)	TSE (% of population)	GDP ($/capita)	PM_10_ (*µ*g/M^3^)	Male smoking rate	Female smoking rate
Asia	9.10 ± 6.78	3.55 ± 2.53	30.23 ± 17.14	10166 ± 12821	87.01 ± 31.18	38.50 ± 9.66	5.10 ± 6.63
Africa	16.43 ± 6.85	5.00 ± 4.36	7.88 ± 2.12	2255 ± 1844	80.01 ± 81.43	31.08 ± 9.68	4.45 ± 4.71
Europe	0.81 ± 0.11	8.93 ± 5.17	57.13 ± 14.03	47323 ± 15284	21.92 ± 3.11	25.86 ± 4.46	22.24 ± 4.20
Miscellaneous	1.49 ± 0.48	9.42 ± 4.21	62.69 ± 28.29	28098 ± 23599	30.70 ± 19.68	20.94 ± 3.65	11.30 ± 6.52

**Table 3:** Correlation among the asthma severity indices and major socioeconomic predictors.

	ARM	Asthma prevalence
TSE	*r* = −0.75 and *p* < 0.001^*∗∗*^	*r* = 0.61, *p* = 0.003^*∗*^
GDP	*r* = −0.68 and *p* = 0.001^*∗*^	*r* = 0.54 and *p* = 0.01^*∗*^

## Data Availability

All the data sources are mentioned in the reference and cited wherever applicable.
